# Can Contemporary Patients with Biopsy Gleason Score 3+4 Be Eligible for Active Surveillance?

**DOI:** 10.1371/journal.pone.0109031

**Published:** 2014-09-30

**Authors:** Ohseong Kwon, Tae Jin Kim, In Jae Lee, Seok-Soo Byun, Sang Eun Lee, Sung Kyu Hong

**Affiliations:** Department of Urology, Seoul National University Bundang Hospital, Seongnam, Korea; Duke University Medical Center, United States of America

## Abstract

**Introduction:**

We analyzed whether expansion of existing active surveillance (AS) protocols to include men with biopsy Gleason score (GS) 3+4 prostate cancer (PCa) would significantly alter pathologic and biochemical outcomes of potential candidates of AS.

**Methods:**

Among patients who underwent radical prostatectomy at our center between 2006 and 2013, we identified 577 patients (group A) who preoperatively fulfilled at least one of 6 different AS criteria. Also, we identified 217 patients (group B) with biopsy GS 3+4 but fulfilled non-GS criteria from at least one of 6 AS criteria. Designating group C as expanded group incorporating all patients in group A and B, we compared risk of unfavorable disease (pathologic GS ≥4+3 and/or pathologic T stage ≥pT3a) and biochemical recurrence (BCR)-free survival between groups.

**Results:**

Rates of unfavorable disease were not significantly different between patients of group A and C who met AS criteria from 5 institutions (all p>0.05), not including University of Toronto (p<0.001). Also BCR-free survivals were not significantly different between patients in group A and C meeting each of 6 AS criteria (all p>0.05). Among group B, PSAD>0.15 ng/mL/cm^3^ (p = 0.011) and tumor length of biopsy GS 3+4 core>4 mm (p = 0.007) were significant predictors of unfavorable disease. When these two criteria were newly applied in defining group B, rates of unfavorable disease in group A and B was 15.6% and 14.7%, respectively (p = 0.886).

**Conclusion:**

Overall rate of pathologically aggressive PCa harbored by potential candidates for AS may not be increased significantly with expansion of criteria to biopsy GS 3+4 under most contemporary AS protocols. PSAD and tumor length of biopsy GS 3+4 core may be useful predictors of more aggressive disease among potential candidates for AS with biopsy GS 3+4.

## Introduction

Currently, active surveillance (AS) is considered as a legitimate alternative to initial radical treatment in the management of low-risk prostate cancer (PCa). Mainly based upon criteria for defining clinically insignificant PCa, different centers have set forth variable selection criteria for AS, mostly incorporating low Gleason score (GS), low clinical stage, and low prostate-specific antigen (PSA) level with estimates of tumor volume from biopsy. In comparison of various criteria for AS, there is a definite trade-off between sensitivity and specificity for prediction of insignificant PCa [1]. Accordingly, stricter criteria would limit the number of men offered AS while excluding some potential candidates. On the other hand, broader criteria would be more prone to cause misclassification of patients with significant disease. It still remains to be answered whether more stringent criteria is better.

Despite varying selection criteria for AS, PCa-specific survival and overall survival rates reported from various AS series have so far been high [2]–[9]. Still, it should be noted that many patients were retrospectively included in these series and did not meet the all the criteria often used in contemporary AS protocol. Although most AS series limit enrollment to biopsy GS 6 tumors, some have described the results of AS in men with biopsy GS 3+4 tumors, demonstrating relatively favorable outcomes [9]. Moreover, some men with higher risk disease may also elect AS in actual clinical setting. However, published data have shown that outcome of GS 3+4 tumors in general, although significantly better than 4+3 tumors, would be worse than GS 6 tumors [10]. Also, the risk of misclassification may increase with the inclusion of GS 3+4 tumors in AS. Although expansion of AS inclusion criteria would contribute to spread benefits of AS to more men, debates continue on the safety of broadening AS criteria to GS 3+4 tumors. Meanwhile, it can be suggested that data on actual pathologic features and prognosis of contemporary radical prostatectomy (RP) patients who preoperatively fulfilled AS criteria except for having biopsy GS 3+4 would prove useful in assessing the oncologic safety of inclusion of such men in AS. Thus, we sought to assess the outcomes of contemporary patients with biopsy GS 3+4 but meeting all other non-GS-related criteria for AS following RP performed as immediate primary treatment for PCa. Using such data, we aimed to analyze whether inclusion of such patients into existing AS protocols would significantly alter the outcomes of AS by comparing the pathologic and biochemical outcomes of potential candidates of AS with or without inclusion of patients with biopsy GS 3+4 tumors. Also we tried to identify useful predictors of unfavorable disease amongst potential AS candidates with biopsy GS 3+4 tumors.

## Patients and Methods

With the approval of the institutional review board at the Seoul National University Bundang Hospital (IRB No.: B-1406/256-111), we reviewed the medical records of 1871 PCa patients who underwent RP at our institution between January 2006 and October 2013. All data were analyzed anonymously. We excluded 385 patients who underwent transrectal ultrasound-guided prostate biopsy obtaining less than 12 cores or who did not have relevant medical record. We then excluded 19 men for whom the period from biopsy to surgery was over 6 months and 33 men who received neoadjuvant hormone therapy. Of the remaining 1434 patients, we identified 577 men (group A) who met at least one of AS selection criteria used by the following institutions: Johns Hopkins Medical Institution (Hopkins) [2], Memorial Sloan-Kettering Cancer Center (MSKCC) [11], Prostate Cancer Research International: Active Surveillance (PRIAS) [3], University of Miami (Miami) [4], University of California, San Francisco (UCSF) [5], and University of Toronto (Toronto) [6]. The criteria implemented by each institution are summarized in Table 1. Additionally, we identified 217 patients (group B) with biopsy GS 3+4 but fulfilled all of other non-GS criteria for AS protocols used by at least one of aforementioned centers. Overall a total of 794 men were included in our study.

**Table 1:** Comparison of contemporary active surveillance protocols.

	Gleason score	PSA (ng/mL)	PSAD (ng/mL/cm^3^)	Number of positive cores	Single core involvement (%)	Clinical stage
Johns Hopkins [2]	≤3+3	-	≤0.15	≤2	≤50	≤cT1c
MSKCC [11]	≤3+3	≤10	-	≤3	≤50	≤cT2a
PRIAS [3]	≤3+3	≤10	<0.2	≤2	-	≤cT2
University of Miami [4]	≤3+3	≤10	-	≤2 (minimum 10 cores)	≤20	≤cT2
UCSF [5]	≤3+3	<10	-	≤33% of all core (of at least 6 cores)	≤50	≤cT2
University of Toronto [6]	≤3+3	≤10	-	-	-	-

MSKCC, Memorial Sloan-Kettering Cancer Center; PRIAS, Prostate Cancer Research International: Active Surveillance;

UCSF, University of California, San Francisco; PSA, prostate-specific antigen; PSAD, prostate-specific antigen density.

For our study, we designated group C as expanded group consisted of all patients in aforementioned group A and B. In all patients, serum PSA levels were examined prior to digital rectal examination and transrectal ultrasound of prostate. Prostate volume was calculated using the prostate ellipsoid formula via transrectal ultrasound of prostate. The TNM stage was recorded according to the 2002 American Joint Committee on Cancer (AJCC) staging system and the GS was reported following the 2005 International Society of Urological Pathology (ISUP) modified scoring system [12]. Unfavorable disease was defined as PCa having pathologic GS ≥4+3 and/or pathologic T stage ≥pT3.

All statistical analyses were performed by commercially available statistical software (IBM SPSS version 19.0, IBM, Armonk, New York, USA). The risk of unfavorable PCa and biochemical recurrence (BCR)-free survival were compared between group A and group C. Clinical parameters were compared by the chi-square test for categorical variables and by Student's t test for continuous variables. The Kaplan-Meier method and the log-rank test were used to assess postoperative BCR-free survival. Within group B, the potential predictors of unfavorable disease were analyzed using multivariate logistic regression models and hazard ratios (HR) and 95% confidence intervals (CI) were calculated. A two-tailed *P* value of <0.05 was considered statistically significant for all statistical analysis.

## Results

Of the 577 patients in group A, 160, 417, 290, 265, 434, and 577 were observed to fulfill AS criteria used in Hopkins, MSKCC, PRIAS, Miami, UCSF, and Toronto, respectively (Table 1). Also, of the 217 patients in group B, 18, 92, 44, 29, 99, and 215 were identified as meeting non-GS AS criteria set forth by Hopkins, MSKCC, PRIAS, Miami, UCSF, and Toronto, respectively. Table 2 shows the clinicopathological profiles of total subjects included in this study. In comparison of group A and B, patients in group B were shown to have significantly higher PSA, PSA density (PSAD), and number of positive cores (all p<0.001). Also group B had significantly higher pathologic GS and stage than group A (both p<0.001). Proportion of unfavorable disease was also higher in group B compared with group A (41.5% vs 15.6%; p<0.001).

**Table 2:** Clinicopathological characteristics of patients.

	Group C (n = 794)	Group A (n = 577)	Group B (n = 217)	P value
Preoperative parameters				
Mean age ± SD (years)	65.4±6.8	65.1±6.9	66.3±6.3	0.014
Mean BMI ± SD (kg/m^2^)	24.3±2.8	24.3±2.6	24.2±3.2	0.602
Clinical Stage, n (%)				0.015
≤T1c	585 (73.7%)	439 (76.1%)	146 (67.3%)	
≥T2a	209 (26.3%)	138 (23.9%)	71 (32.7%)	
Mean prostate volume ± SD (cm^3^)	36.8±14.8	37.7±15.3	34.5±13.4	0.007
Mean PSA ± SD (ng/mL)	5.8±2.1	5.6±2.0	6.2±2.1	<0.001
Mean PSA density ± SD (ng/mL/cm^3^)	0.18±0.09	0.17±0.09	0.20±0.09	<0.001
No. of biopsy cores, n (%)				0.170
12	562 (70.8%)	418 (72.4%)	144 (66.4%)	
13	99 (12.5%)	65 (11.3%)	34 (15.7%)	
≥14	133 (16.7%)	94 (16.3%)	39 (18.0%)	
No. of positive core, n (%)				<0.001
1	277 (34.9%)	240 (41.6%)	37 (17.1%)	
2	171 (21.5%)	130 (22.5%)	41 (18.9%)	
≥3	346 (43.6%)	207 (35.9%)	139 (64.1%)	
Mean tumor length in positive core ± SD (mm)	4.1±3.0	3.4±2.7	5.7±3.1	<0.001
Mean tumor length in 3+4 core ± SD (mm)	-	-	5.3±3.1	-
Postoperative parameters				
Pathological Gleason score, n (%)				<0.001
6	184 (23.2%)	182 (31.5%)	2 (0.9%)	
7 (3+4)	518 (65.2%)	353 (61.2%)	165 (76.0%)	
7 (4+3)	83 (10.5%)	36 (6.2%)	47 (21.7%)	
≥8	9 (1.1%)	6 (1.0%)	3 (1.4%)	
Pathological Stage, n (%)				<0.001
≤T2c	682 (85.9%)	521 (90.3%)	161 (74.2%)	
≥T3a	112 (14.1%)	56 (9.7%)	56 (25.8%)	
Positive surgical margin, n (%)	140 (17.6%)	90 (15.6%)	50 (23.0%)	0.016
Unfavorable prostate cancer, n (%)	180 (22.7%)	90 (15.6%)	90 (41.5%)	<0.001

BMI, body mass index; PSA, prostate specific antigen.

By applying each of 6 different AS criteria from 6 aforementioned institutions, patients meeting these criteria in group A and group C were compared with regards to the rates of unfavorable disease and BCR-free survival (Table 3). The rates of unfavorable disease were not significantly different between patients in group A and C who met criteria from each of 5 following institutions (Hopkins, MSKCC, PRIAS, Miami, and UCSF; p = 0.564, 0.073, 0.262, 0.571, and 0.077, respectively). Only patients in group C fulfilling criteria from Toronto had a significantly higher rate of unfavorable disease than counterparts in group A (p = 0.001). On the other hand, BCR-free survivals were not demonstrated to be significantly different between patients in group A and C who met each of 6 criteria (Hopkins, MSKCC, PRIAS, Miami, UCSF, and Toronto; p = 0.972, 0.890, 0.811, 0.927, 0.872, and 0.915, respectively). The median follow-up time was 38.5 months (interquartile range, 19.0–56.0).

**Table 3:** Comparison of the rates of unfavorable disease and the 5-year biochemical recurrence free survivals between patients in group A and C fulfilling each active surveillance protocol.

Protocols	Group A	Group C	P value
Johns Hopkins			
Unfavorable disease, n (%)	10 (6.3%)	14 (7.9%)	0.564
5-year BCR free survival (%)	97.9%	98.0%	0.972
MSKCC			
Unfavorable disease, n (%)	55 (13.2%)	89 (17.5%)	0.073
5-year BCR free survival (%)	97.6%	97.7%	0.890
PRIAS			
Unfavorable disease, n (%)	29 (10.0%)	43 (12.9%)	0.262
5-year BCR free survival (%)	97.0%	97.3%	0.811
University of Miami			
Unfavorable disease, n (%)	25 (9.4%)	32 (10.9%)	0.571
5-year BCR free survival (%)	97.3%	97.5%	0.927
UCSF			
Unfavorable disease, n (%)	57 (13.1%)	92 (17.3%)	0.077
5-year BCR free survival (%)	96.6%	96.8%	0.872
University of Toronto			
Unfavorable disease, n (%)	90 (15.6%)	179 (22.6%)	0.001
5-year BCR free survival (%)	93.8%	93.8%	0.915

MSKCC, Memorial Sloan-Kettering Cancer Center; PRIAS, Prostate Cancer Research International: Active Surveillance; UCSF, University of California, San Francisco; BCR, biochemical recurrence.

Analyzing patients in group B, multivariate analysis was performed to identify useful predictor of unfavorable disease in group B (Table 4). Among the group B patients who met AS criteria from Hopkins or Miami, no significant predictor of unfavorable disease was revealed for group B. However, among men who met one of other four criteria, PSAD>0.15 ng/mL/cm^3^ (MSKCC, PRIAS, UCSF, and Toronto; p = 0.024, 0.035, 0.028, and 0.012, respectively) and the tumor length in biopsy GS 3+4 core>4 mm (MSKCC, PRIAS, UCSF, and Toronto; p = 0.025, 0.032, 0.022, and 0.008, respectively) were observed to be significant predictors for unfavorable disease. When analyzing all the patients in group B, PSAD>0.15 ng/mL/cm^3^ (HR 2.273, 95% CI 1.207–4.283, p = 0.011) and tumor length of biopsy GS 3+4 core>4 mm (HR 2.338, 95% CI 1.261–4.337, p = 0.007) were also found to be significant predictors of unfavorable disease. When these two criteria of PSAD and tumor length of biopsy GS 3+4 core were newly applied in defining group B, the number of total patients included in group B decreased from 217 to 34. Also the rate of unfavorable disease in group B decreased from 41.5% to 14.7% which was not significantly different with aforementioned rate of 15.6% observed in group A (p = 0.886).

**Table 4:** Multivariate analysis of parameters predicting pathologically unfavorable prostate cancer among patients in group B fulfilling each active surveillance protocol.

Protocols		Age, years>65 vs ≤65	BMI, kg/m^2^>24 vs ≤24	Clinical T ≥T2a vs ≤T1c	No. of GS 3+4 core ≥2 vs single	PSAD, ng/mL/cm^3^>0.15 vs ≤0.15	Tumor length in a GS 3+4 core, mm>4 vs ≤4
Overall Group B	HR	1.281	1.452	0.875	0.799	2.273	2.338
	95% CI	0.711–2.308	0.817–2.581	0.470–1.628	0.433–1.476	1.207–4.283	1.261–4.337
	P value	0.409	0.204	0.674	0.474	0.011	0.007
Johns Hopkins	HR	0.183	1.931	-	0.000	-	3.935
	95% CI	0.007–4.681	0.112–33.174	-	0.000-	-	0.147–105.357
	P value	0.304	0.650	-	0.999	-	0.414
MSKCC	HR	0.633	1.450	0.514	0.339	3.415	3.776
	95% CI	0.245–1.634	0.551–3.814	0.148–1.789	0.094–1.225	1.177–9.911	1.183–12.056
	P value	0.344	0.452	0.296	0.099	0.024	0.025
PRIAS	HR	0.226	0.593	0.465	0.046	7.383	11.419
	95% CI	0.044–1.162	0.109–3.224	0.081–2.662	0.003–0.756	1.152–47.296	1.227–106.240
	P value	0.075	0.545	0.390	0.031	0.035	0.032
University of Miami	HR	0.344	0.314	0.000	0.000	1.322	4.864
	95% CI	0.029–4.025	0.038–2.557	0.000-	0.000-	0.113–15.449	0.000-
	P value	0.395	0.279	0.999	1.000	0.824	1.000
UCSF	HR	0.795	1.566	0.419	0.312	3.208	3.615
	95% CI	0.320–1.976	0.620–3.959	0.129–1.358	0.087–1.119	1.136–9.061	1.203–10.864
	P value	0.621	0.343	0.147	0.074	0.028	0.022
University of Toronto	HR	1.276	1.448	0.879	0.803	2.286	2.326
	95% CI	0.706–2.309	0.813–2.579	0.470–1.644	0.432–1.493	1.204–4.341	1.245–4.345
	P value	0.420	0.208	0.687	0.489	0.012	0.008

MSKCC, Memorial Sloan-Kettering Cancer Center; PRIAS, Prostate Cancer Research International: Active Surveillance; UCSF,

University of California, San Francisco.

## Discussion

In this study of patients deemed as potential AS candidates according to several contemporary AS protocol but who underwent RP as immediate primary treatment for PCa, we found that the rate of unfavorable disease was not significantly altered for most of AS protocols when subjects were expanded to include patients with biopsy GS 3+4 fulfilling other non-GS selection criteria for AS. Also postoperative BCR-free survivals, though follow-up durations were modest, were observed to be comparable even with inclusion of patients with biopsy GS 3+4. As a proportion of patients fulfilling contemporary selection criteria for AS is expected to harbor unfavorable disease, our findings suggest that expansion of most AS selection criteria to biopsy GS 3+4 would not result in a significant increase of such misclassification of worse disease when analyzing potential candidates for AS as a whole. Furthermore, although the rates of unfavorable pathologic features would certainly be higher in patients with GS 3+4 than those with GS 3+3, we observed that when additional parameters, such as PSAD and tumor length of biopsy GS 3+4 core, are applied to potential candidates for AS but having biopsy GS 3+4, the rate of unfavorable disease among such candidates would be significantly decreased being comparable to the level without expansion of criteria to GS 3+4. Overall our finding would provide support to the idea of expanding the benefits to AS to more patients.

The outcomes of AS series are being reported by several groups with increasing duration of follow-up [2], [3], [6]. In the Johns Hopkins AS series, median follow-up duration being 2.7 years (range, 0.01 to 15.0 years), overall survival and cancer specific survival rates were 97% and 100%, respectively [2]. From PRIAS study, 4-year overall survival rate was reported to be 86.5% with no cancer specific death [3]. In the Toronto series, which is the most mature, the overall survival rate was 78.6% and the 10-year PCa actuarial survival was 97% [6]. Accordingly, available data indicate AS is indeed a safe and effective approach of management for low-risk PCa. Meanwhile, there are AS series which included patients with higher risk PCa [13]–[15]. Cooperberg et al. showed that progression-free survival at 4 years among patients with intermediate-risk PCa (61%) was not significantly different from low-risk patients (54%) on AS [13]. In their series, 27 of 90 intermediate risk patients (30%) had biopsy GS 3+4. Also, the medium-term outcomes of AS from Royal Marsden Hospital demonstrated that 5-year treatment free rate was 70% (95% CI 65-75%) and the PCa-specific deaths occurred in only 2 of 471 patients [14]. In this series, 33 of 471 (7%) total subjects had GS 3+4, which included one of the two patients expired due to PCa. Bul et al. also showed the potential expandibility for AS including intermediate risk patients [15]. Their AS series was composed of low-risk patients who fulfilled the contemporary PRIAS criteria and intermediate-risk patients with PSA 10 to 20 ng/mL or GS 7 or three positive cores. After median follow-up of 7.4 years, the 10-year cancer specific survival rates were 99.1% for low risk patients and 96.1% for intermediate risk patients (p = 0.44) in their series [15]. Overall published reports have so far demonstrated that AS can be a feasible treatment approach in selected group of men with intermediate-risk PCa.

As prostate size has been reported to be significantly related with Gleason upgrading in several studies, PSAD has been mentioned as being a more accurate predictor of upgrading than PSA alone [16]–[18]. Davies et al. suggested that enlarging prostate size was significantly associated with less incidence of any GS upgrading and major GS upgrading [16]. Also, Turley et al. reported that a smaller prostate was an independent predictor of GS upgrading [17]. In a study of Magheli et al., PSAD was shown to be a significant predictor of Gleason upgrading in multivariate analysis while PSA level lost its predictive power [18]. In the current study, PSAD was observed as an independent predictor of unfavorable PCa among the patients with biopsy GS 3+4. Although not all AS protocols incorporated PSAD as a part of selection criteria for AS candidates, our findings demonstrated that PSAD can be useful when considering expanding AS criteria.

The percentage or length of cancer involvement in a single biopsy core has also been mentioned as a potentially useful tool for the patient selection in AS [19]. Russo et al. reported that the percentage of cancer involvement in biopsy cores was a predictor of unfavorable PCa among patients treated with RP but eligible for AS according to PRIAS criteria [20]. In their study, cancer involvement <4 mm was proposed as a predictor for decreased risk of biochemical recurrence. Also, Yashi et al. suggested that biopsy tumor length less than 2 mm was an independent factor predicting insignificant cancer [21]. In a study by Freeland et al., the percentage of tissue with cancer was a significant predictor of unfavorable PCa and biochemical recurrence after RP [22]. They found that tumor percentage less than 20% predicted a decreased risk of BCR after RP. In our study, the tumor length in a biopsy GS 3+4 core was identified as a independent predictor of unfavorable PCa among men with biopsy GS 3+4. We observed that a cutoff value of 4 mm was a significant predictor of unfavorable PCa in such group of patients. Accordingly, we believe that tumor length in a biopsy GS 3+4 core, in addition to PSAD, would prove useful in safely expanding AS criteria to men with biopsy GS 3+4.

When comparing the 6 AS criteria analyzed in the current study, the criteria from University of Toronto can be considered the most inclusive one. In case of Toronto criteria, the discriminating ability to predict favorable PCa was suboptimal due to its high sensitivity and low specificity [1]. For such reason, unlike other 5 criteria, the expansion of Toronto criteria to men with biopsy GS 3+4 probably contributed to significant increase in unfavorable pathology. On the other hand, the John Hopkins criteria was the most stringent criteria as the number of our subjects eligible was the lowest among the 6 criteria. The Miami criteria can also be considered as being one of more strict criteria, incorporating tumor length less than 20% in a single positive biopsy core. As Hopkins and Miami criteria were relatively more stringent, already incorporating PSAD or tumor length, no significant predictor of unfavorable disease among potential AS candidates with GS 3+4 was identified among conventional variables when applying the two criteria. Meanwhile as PSAD and tumor length in GS 3+4 core proved to be a significant predictor for unfavorable disease among the group B as a whole in this study, we believe that the application of these two parameters would be helpful in the expansion of AS criteria by minimizing the risk of misclassification.

There are new approaches to improve the ability to predict biopsy reclassification. Many studies have been reported on the value of magnetic resonance imaging (MRI) in AS [23]–[26]. Published reports have shown that multiparametric MRI generally demonstrates a very high negative predictive value for upgrading. Accordingly, favorable MRI findings may be used for selection and follow-up of patients during AS, possibly obviating the need for repeat biopsies. In addition, Song et al. reported that multiparametric MRI even without dynamic contrast enhanced MRI significantly helped to predict GS upgrading or pathologic upstaging after RP in low risk PCa patients [26]. Meanwhile, a host of biomarkers have been suggested to augment AS programs [27]. Several studies have been conducted to examine gene expression signatures in prostate biopsies. A cell cycle gene expression array has been used to predict risk of disease progression and to recommend additional treatment for corresponding patients [28]. A 17-gene assay has been found to be associated with risk of clinical recurrence and high grade disease [29]. New imaging and biomarkers may improve future patient selection and follow-up in AS. Prospective studies should aim to evaluate new tools in AS setting.

Limitations of this study would include its retrospective design. Also, only men who underwent RP were included in this study. In addition, postoperative follow-up durations of our subjects were relatively short. However, it should be noted that all of our subjects underwent extended prostate biopsy obtaining minimum of 12 cores. Also GS was graded according to new modified ISUP scoring system in our contemporary cohort of subjects.

## Conclusion

In this study, our findings suggested that the actual rate of pathologically aggressive PCa harbored by potential candidates for AS as a whole would not significantly increase with expansion of selection criteria to biopsy GS 3+4 under most contemporary AS protocols. Although GS 3+4 tumors would have worse pathologic features than GS 3+3 tumors even among potential candidates for AS, additional parameters of PSAD and tumor length in biopsy GS 3+4 may prove useful in the identification of appropriate candidates for AS among biopsy GS 3+4 group. We believe that our findings warrant further investigations to develop feasible and safe way to expand the selection criteria for AS as well as provide additional support to the idea of expanding the benefits to AS to more patients.
